# Synonymous Variants of Potential Significance Identified by a 52‐Gene Clinical Sequencing Panel in Non‐Small Cell Lung Cancer

**DOI:** 10.1002/gcc.70111

**Published:** 2026-02-19

**Authors:** Kathleen Varty, Leah MacLean, Doha Itani, Monowar Hossain, Cenk Acar, James Michael, Trisha Daigle‐Maloney, Robert Thompson, Brian Johnston, Crispin Russell, Jeanette E. Boudreau, Daniel Gaston, Tony Reiman

**Affiliations:** ^1^ Department of Biological Sciences University of New Brunswick Saint John New Brunswick Canada; ^2^ Beatrice Hunter Cancer Research Institute Halifax Nova Scotia Canada; ^3^ Department of Medicine Dalhousie University Saint John New Brunswick Canada; ^4^ Department of Pathology Dalhousie University Halifax Nova Scotia Canada; ^5^ Department of Pathology Dalhousie University Saint John New Brunswick Canada; ^6^ Department of Pathology Saint John Regional Hospital Saint John New Brunswick Canada; ^7^ Department of Oncology Saint John Regional Hospital Saint John New Brunswick Canada; ^8^ Department of Radiation Oncology Dalhousie University Saint John New Brunswick Canada; ^9^ Division of Thoracic Surgery Saint John Regional Hospital Saint John New Brunswick Canada; ^10^ Department of Surgery Dalhousie University Saint John New Brunswick Canada; ^11^ Department of Microbiology and Immunology Dalhousie University Halifax Nova Scotia Canada; ^12^ Department of Pathology and Laboratory Medicine Nova Scotia Health Authority Halifax Nova Scotia Canada

## Abstract

**Introduction:**

Next‐generation sequencing (NGS) is routinely used for lung cancer genomic profiling to identify known, actionable, non‐synonymous driver mutations. Recent findings suggest that synonymous variants may also be cancer drivers.

**Materials and Methods:**

We analyzed genomic data and clinical outcomes for patients (*n* = 353) with non‐small cell lung cancer (NSCLC) sequenced with the Oncomine Focus 52‐gene NGS panel (ThermoFisher Scientific, Waltham, MA, USA) at the Saint John Regional Hospital in New Brunswick, Canada, from January 2019 to January 2023.

**Results:**

KRAS was the most commonly mutated gene in this cohort from Saint John, New Brunswick, with a higher prevalence than reported in other populations. Several novel synonymous variants were identified, including in *ALK*, *EGFR, FGFR2*, *FGFR3*, *MYC, NF1, NRAS*, and *PIK3CA*, with potential effects on MAPK/ERK and PI3K/AKT signaling.

**Conclusion:**

This cohort demonstrates both expected and distinct genomic features, including novel synonymous variants in oncogenic pathways. These findings suggest regional variation in NSCLC genomics and support further study of synonymous variants in disease progression.

## 1 Introduction

With an estimated 1.76 million deaths per year, lung cancer is the leading cause of cancer‐related deaths, both in Canada and worldwide [1, 2]. The Maritime Canadian provinces (New Brunswick, Nova Scotia, Prince Edward Island) generally have higher lung cancer incidence and mortality rates compared to the rest of Canada [2]. The increased lung cancer burden in this region is driven by several factors including an older population, an increased prevalence of smoking, and increased exposure to the environmental and occupational lung carcinogens, radon and arsenic [3, 4, 5, 6].

Accounting for 85% of all lung cancers, non‐small cell lung cancer (NSCLC) is the most prevalent subtype [7]. The standard of care in Canada is to screen all non‐squamous NSCLC for actionable mutations to inform personalized therapies [8, 9]. Most of the research into genetic drivers of NSCLC has focused on non‐synonymous mutations: mutations that result in a change in the encoded protein sequence [10]. Recent evidence suggests that synonymous mutations—those found in protein coding DNA regions but do not result in a change in the encoded protein sequence—may also have an oncogenic impact like known non‐synonymous driver mutations [11, 12]. This emerging evidence broadens our understanding of oncogenesis, emphasizing the need to reassess the functional significance of synonymous mutations in cancer biology. Although no change occurs in the protein form, synonymous mutations can influence the availability and efficiency of the gene product by impacting the speed and accuracy of mRNA translation, folding, and splicing [12, 13, 14]. For instance, while the exact mechanism is unknown, a synonymous mutation in *IDH1* is an adverse prognostic factor in acute myeloid leukemia [15]. The prevalence and possible clinical implication of synonymous mutations in lung cancer and cancer more broadly is under‐investigated [16].

Previous studies examining targeted sequencing panels in lung cancer have primarily focused on established driver and clinically actionable mutations [17, 18], without examining the rich additional sequencing data generated by these panels. There is also a paucity of data on the genomic characteristics of NSCLC cases arising in Atlantic Canada. Here we address these gaps by performing a comprehensive analysis of clinical NSCLC Next‐Generation Sequencing (NGS) data at an Atlantic Canadian centre, including synonymous mutations and their relation to clinical outcomes and clinical markers.

## 2 Materials and Methods

### 2.1 Study Design and Descriptive Statistics

The project was reviewed by Horizon Health Network Research Ethics Board (RS #: 2023‐3215, approval date 8 February 2023) and the University of New Brunswick (Saint John) Research Ethics Board (File #: 2023‐046, approval date 8 May 2023). Patients' consent was waived due to the retrospective nature of the study and publication of aggregate and anonymized data only. This retrospective case series analyzed genomic and clinical data initially collected for clinical use. The study included patients diagnosed with NSCLC who underwent a 52‐gene NGS panel at the Saint John Regional Hospital (SJRH) between January 2019 and January 2023. At this hospital, all non‐squamous NSCLC specimens are routinely subjected to NGS testing, as per the guidelines from the National Comprehensive Cancer Network [19], regardless of disease stage. Of the 353 patients included, nine were excluded from the demographic analysis due to incomplete chart information. The following clinical variables were collected from patient electronic medical charts: sex, location of care, cancer diagnosis and staging, variants detected from gene panel, treatment received, age at diagnosis, smoking history, length of OS, and length of PFS. The clinical and demographic variables obtained from medical records were as follows: sex, location of care, date of birth, cancer diagnosis and TNM staging, variants detected from gene panel, treatment received, last date known alive or date of death, date of diagnosis, date genetic panel received, the date of first cancer progression following diagnosis (if applicable), and smoking status.

Smoking status was divided into three categories: never‐smoker, former smoker, and current smoker. Never smoker describes someone who has never smoked or smoked less than 100 cigarettes in their life. Former smokers describe those who smoked 100 or more cigarettes in their lifetime but had stopped smoking at least 1 month or longer before their diagnosis. Current smokers were smoking upon diagnosis or stopped less than 1 month before their diagnosis [20, 21, 22]. The proportion of patients deceased is likely an underestimate as mortality status could not unequivocally be determined for all patients (ex. no obituary is available) [23].

Descriptive statistics for the cohort were determined using Prism [24]. Categorical variables collected were as follows: sex, location of care, cancer diagnosis and staging [25], variants detected from gene panel, treatment received, and smoking status. Continuous variables included age at diagnosis, length of overall survival (OS), and length of progression‐free survival. Only patients with accessible medical records could be included in the descriptive statistics for the cohort. Length of progression‐free and overall survival was measured in days. Time zero was defined as the date that tissue was first obtained which led to a diagnosis of NSCLC. The end point for OS was the date of death. The end point for PFS was the date the patient experienced either disease progression or death (whichever was first documented). Health records and publicly available obituaries were reviewed for each patient to ascertain their mortality status and the date of their passing if applicable. For those patients who had not yet reached the PFS or OS endpoint, data were censored at the date last known to be alive as determined by the patient's last documented visit to the hospital. Progression was determined from diagnostic imaging reports, and as documented in the treating physician's notes.

### 2.2 Tumor Sample Processing and Sequencing

Sequencing was performed by the Molecular Diagnostics and Cytogenetics division in the Pathology department at the Saint John Regional Hospital. All genetic data used were initially sequenced and processed using the IonTorrent Oncomine Focus Assay (ThermoFisher Scientific, Waltham, MA, USA), an NGS platform [26]. A tumor‐only sequencing approach was used. Samples were analyzed for clinical reporting with Ion Reporter 5.6 and its 5.6 analysis workflow until the end of 2021, after which, starting in January 2022, analysis was conducted using Ion Reporter 5.18 and its 5.18 analysis workflow. IonTorrent Oncomine Focus Assay (ThermoFisher Scientific, Waltham, MA, USA) covers 52 genes related to cancer for both SNV and CNVs. This sequencing produces a raw sequencing, Binary Alignment Map (BAM), and Variant Call Format (VCF) file. The VCFs collected from the clinical server were the starting point for the analysis.

### 2.3 VCF Processing

All genomic data were de‐identified by assigning a randomly generated 6‐digit identifier to each patient. The variants in each VCF were then sorted by genomic coordinate before annotations. Next, the files were converted from reference genome GRCh37 to the GRCh38 assembly using CrossMap (v0.6.6) [27]. The VCFs were decomposed and normalized using bcftools (v1.8) [28].

### 2.4 Variant Annotation and Filtering

The input for our analysis was all variants that passed Ion Torrent's in‐house quality filtering. Finally, variants with a VAF of less than 2.5% were removed. The threshold of 2.5% aligns with the standard used in the Molecular Diagnostics and Cytogenetics division in the Pathology department at the Saint John Regional Hospital for clinical reporting.

Additional annotations were added to each variant transcript using Ensembl's VEP (release 111). The Docker software platform was used to run VEP and four plugins were utilized: dbNSFP (v4.6), dbscSNV (v1.1), SpliceRegion (v2.0), and SpliceAI (v1.3). For pathogenicity predictions, the web interfaces CScape and TRaP (v3.0) were employed [29, 30, 31, 32].

Matched Annotation from NCBI and EMBL‐EBI (MANE) transcripts were selected for annotation and to predict variant effects [33]. Annotations from both NCBI and EMBL‐EBI for the same MANE transcript were merged to retain all relevant annotations.

To remove population associated variants, common single nucleotide polymorphisms (SNPs) with an allele frequency greater than 0.5% in the Genome Aggregation Database (gnomAD) were removed [34].

Given the exploratory nature of the study, germline variants were conservatively filtered out by removing variants with a VAF of 50% or 95%–100% unless the variant had an annotation in COSMIC. Any variant appearing in > 20% of the cohort or found to have a significant impact on survival was reviewed using the Broad Institute's Integrative Genomics Viewer (IGV) to identify any signatures that may indicate that a variant is an artifact or polymorphism [35]. Additionally, any variant with significant predictions of pathogenicity or splicing impact was reviewed.

## 3 Results

A total of 353 patients were included in the study. Nine patients with incomplete chart information were excluded from the demographic analysis (age at time of diagnosis, sex, smoking status, subtype, and TNM stage) but included in the genomic analysis. The age at diagnosis ranged from 28 to 89 years, with a mean of 68 ± 8.5 years (SD). Females were slightly more prevalent in the cohort (52.90%). The three most common subtypes within our cohort of NSCLC patients were adenocarcinoma (72.97%), squamous cell carcinoma (17.73%), and poorly differentiated NSCLC (6.69%) (Table 1).

**TABLE 1:** Cohort demographic.

Characteristic	Category	Frequency (N)	Percentage (%)	Mean +/− SEM	Range
Age				68+/−0.45	28–89
Sex	Male	162	47.1		
	Female	182	52.9		
Smoking status	Current smoke	130			
	Former smoker	170			
	Never smoker	43			
	Unknown	1			
Subtype	Adenocarcinoma	251	73.0		
	Squamous cell carcinoma	61	17.7		
	Poorly differentiated NSCLC	23	6.69		
	Large cell carcinoma	5	1.45		
	Adenocarcinoma and squamous cell carcinoma	2	0.581		
	Carcinoid tumor	1	0.291		
	Adenosquamous carcinoma	1	0.291		
AJCC 8th edition TNM stage	I	131	38.1		
	II	26	7.56		
	III	58	16.9		
	IV	125	36.3		
	Unknown	4	1.16		

Most patients were diagnosed at TNM stage I (38.1%) or IV (36.3%) and four patients were unable to be staged. Radiation, surgical resection, and chemotherapy were the three most common treatments received. Smoking status was considered as three categories: never‐smoker, former smoker, and current smoker. Never smoker describes someone who has never smoked or smoked fewer than 100 cigarettes in their lifetime. Former smokers include those that have smoked 100 or more cigarettes in their lifetime but have stopped smoking at least 1 month prior to their diagnosis. Current smokers were smoking upon diagnosis or stopped less than 1 month before their diagnosis [20, 21, 22]. Most patients were either former smokers (49.56%) or current smokers (37.90%).

**FIGURE 1:**
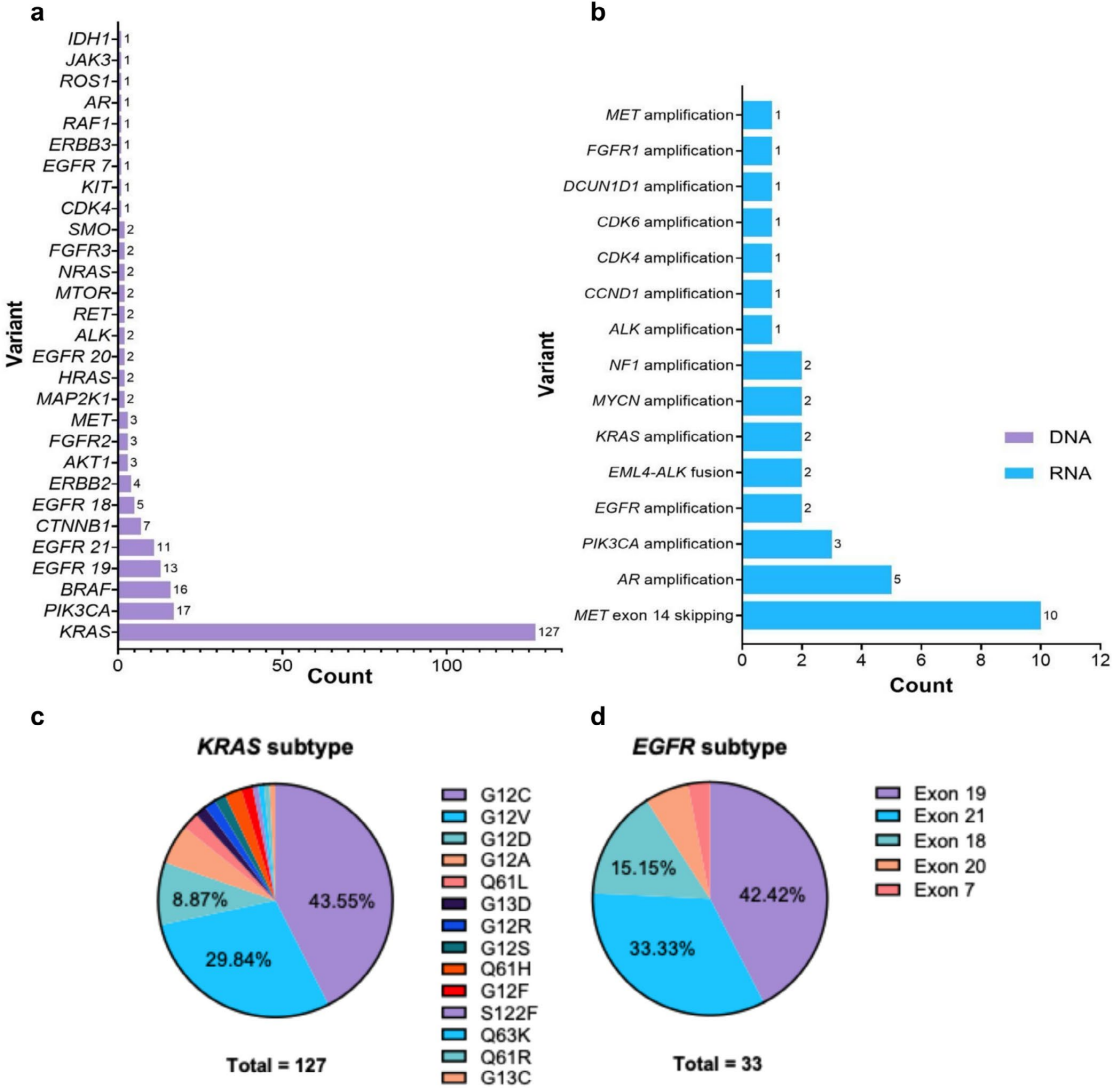
Clinically reported variants in cohort (*n* = 353) that were defined using the 52‐gene panel. (a) Total number of clinically reported DNA variants per gene. Counts reflect all reported variants, not unique patients (i.e., one patient may have more than one variant present; 267 total variants are reported for 220 patients in whom mutations were found). (b) Total number of clinically reported RNA fusion events per gene, with the same counting approach as in (a). (c) Distribution of KRAS mutation subtypes among the 124 reported variants. (d) Distribution of EGFR mutation subtypes among the 33 reported variants.

At the time of data collection, 63.7% (*n* = 219) of patients had reached the progression‐free survival (PFS) endpoint. Of these, 43.9% (*n* = 155) of patients exhibited disease progression and 48.7% (*n* = 168) of patients were deceased. Of the 353 patients included in the genomic analysis, 62.3% had at least one clinically reported non‐synonymous variant, with an average of approximately one variant per patient among those affected (1.26 ± 0.64). This aligns with previous studies, which have identified driver mutations in approximately 60% of adenocarcinomas [12]—the predominant histological subtype in this study. The most common clinically reported variants detected with DNA sequencing were found in *KRAS*, *PIK3CA*, and *BRAF*. Among variants detected with RNA sequencing, *MET* exon 14 skipping, *AR* amplification, and *EGFR* amplification were most prevalent (Figure 1). Compared to existing literature, the prevalence of the *KRAS* G12V subtype in this cohort is approximately 10% greater [17, 36, 37].

A total of 311 novel—defined as not established in the literature, COSMIC [38, 39], or ClinVar [40] as associated with NSCLC variants—were identified at time of submission; these occurred 366 times in the cohort with each patient possessing at least one. Of these 311 variants, 53 were synonymous, and these occurred a combined 67 times in 45 patients. Two of these synonymous variants—one in *BRAF* and one in *NRAS*—were among the 10 most prevalent unique variants in the cohort, occurring in 12 patients.

Of the 353 patients, 148 (41.9%) had at least one novel variant. Across all novel variant calls (*n* = 364), the VAFs ranged from 0.025 to 0.8905 (average 0.1980 ± 0.2037). For recurrent novel variants observed in multiple patients, VAFs ranged between individuals. The cohort exhibited considerable diversity, with most patients harboring a single variant. Three patients had a very high number of variants, with 99, 31, and 25 novel variants identified.

We next used SpliceAI [41] to interrogate these novel variants for potential impacts on splice sites and identified 25 synonymous variants. The G to A substitution in *FGFR3* (chr4:1804418 G>A, *n* = 1) was most likely to impact splicing as indicated by SpliceAI, with an acceptor loss score of 0.44, which indicates that the substitution may prevent the normal recognition and use of the acceptor site during splicing. The next synonymous variant most likely to impact splicing was a C to T substitution in *MYC* (chr8:127738274 C>T) with a SpliceAI donor loss score of 0.19, slightly below the recommended threshold. Similarly to the acceptor, the donor loss suggests the variant may prevent the normal functioning of the donor site during splicing. Neither the *FGFR3* mutation nor the *MYC* mutation co‐occurred with established clinically reported variants in their respective genes.

We next used CScape to identify potentially oncogenic mutations [29]. CScape assigns a probability score to each SNV indicating the likelihood that the variant is cancer‐associated and pathogenic. CScape scores range from 0 to 1, with higher scores indicating a greater model‐estimated probability that the variant functions as an oncogenic driver. The model was trained using known somatic driver and passenger mutations from curated cancer datasets. Five synonymous variants were identified as potentially oncogenic, each with a low confidence score, suggesting this result requires further validation and investigation (Table 2). The *NRAS* variant—identified as potentially pathogenic by CScape—was part of the top 10 most prevalent variants in the cohort.

**TABLE 2:** Synonymous variants with possible pathogenicity in cancer as defined by CScape score > 0.5. Scores for additional predictive algorithms, occurrence, and allele frequency or range are also included.

Gene	Variant	Cscape			TraP score	CADD Phred‐like score	Occurrence	Allele frequency/range
		Score	Prediction	Confidence				
*ALK*	chr2:29213992 G>A	0.656541	Oncogenic	Low	0.131	11.45	1	0.0440613
*FGFR2*	chr10:121515246 G>A	0.641029	Oncogenic	Low	0.104	12.63	1	0.0643821
*NF1*	chr17:31358501 G>A	0.667882	Oncogenic	Low	0.104	8.931	1	0.0566038
*NRAS*	chr1:114713886 T>C	0.505196	Oncogenic	Low	0.009	12.59	4	0.0503597–0.0715686
*PIK3CA*	chr3:179204568 A>G	0.520249	Oncogenic	Low	0.027	12.35	1	0.162

The second algorithm utilized was the Transcript‐inferred Pathogenicity (TraP) score [30]. Like CScape, the TraP score ranges from 0 to 1 with higher scores indicating a greater likelihood that a variant will disrupt transcript structure or function and therefore have a greater pathogenic potential. TraP uses a machine learning model trained on known pathogenic variants and common benign variants. Eleven variants were identified with a TraP score greater than 0.100. TraP scores are interpreted on a percentile scale; 0.100 was chosen because it is between the 50 and 75th percentiles. Two variants—one in *SMO* and the other in *FGFR3—*had very high scores in the 0.400 range indicating a great likelihood to be disease causing. All other variants had a TraP score in the low 0.100 range (Table 3).

**TABLE 3:** Synonymous variants with potential pathogenicity as denoted by TraP score > 0.100. Scores for additional predictive algorithms, occurrence, and allele frequency or range are also included.

Gene	Variant	Cscape			TRaP score	CADD Phred‐like score	Occurrence	Allele frequency/range
		score	Prediction	Confidence				
*SMO*	chr7:129209384 C>A	0.261754	Neutral	Low	0.429	12.79	1	0.0460526
*FGFR3*	chr4:1804418 G>A	0.148019	Neutral	Low	0.427	18.47	1	0.37302
*MYC*	chr8:127738274 C>T	0.159967	Neutral	Low	0.131	12.48	1	0.626
*ALK*	chr2:29213992 G>A	0.656541	Oncogenic	Low	0.131	11.45	1	0.0440613
*ERBB3*	chr12:56085090 C>G	0.239366	Neutral	Low	0.123	9.763	1	0.332666
*EGFR*	chr7:55191841 G>A	0.188506	Neutral	Low	0.114	2.405	2	0.047–0.488924
*SMO*	chr7:129210486 C>A	0.272064	Neutral	Low	0.105	5.765	1	0.234281
*FGFR2*	chr10:121515246 G>A	0.641029	Oncogenic	Low	0.104	12.63	1	0.0643821
*NF1*	chr17:31358501 G>A	0.667882	Oncogenic	Low	0.104	8.931	1	0.0566038
*ALK*	chr2:29220757 G>A	0.274993	Neutral	Low	0.103	3.457	1	0.635135
*EGFR*	chr7:55181331 G>A	0.158837	Neutral	Low	0.101	15.34	1	0.0503751

The Combined Annotation Dependent Depletion (CADD) score [42, 43] was the final prediction score used. Higher scores indicate a greater likelihood that the variant is functionally significant and potentially deleterious. The CADD score, which can be represented on a logarithmic scale, is commonly referred to as a CADD Phred‐like score. The authors recommend a ranking system rather than a hard cut‐off but an explanation of score is available. A CADD Phred‐like score of 10 or greater indicates that the variant analyzed is predicted to be in the 10% most deleterious substitutions possible in the human genome. A total of 20 synonymous variants were identified with a CADD Phred‐like score of 10 or greater; this included four of the variants identified as potentially oncogenic using CScape (Table 4). The variant with the highest CADD Phred‐like score was a G to A substitution in *FGFR3*.

**TABLE 4:** Synonymous variants with a CADD Phred‐like score > 10. Scores for additional predictive algorithms, occurrence, and allele frequency or range are also included.

Gene	Variant	Cscape			TRaP score	CADD Phred‐like score	Occurrence	Allele frequency/range
		Score	Prediction	Confidence				
*FGFR3*	chr4:1804418 G>A	0.148019	Neutral	Low	0.427	18.47	1	0.37302
*FGFR3*	chr4:1801857 C>T	0.271777	Neutral	Low	0.068	16.03	1	0.242424
*BRAF*	chr7:140794401 T>C	0.203643	Neutral	Low	0.056	15.81	1	0.0554562
*EGFR*	chr7:55181331 G>A	0.158837	Neutral	Low	0.101	15.34	1	0.0503751
*ERBB2*	chr17:39725096 C>T	0.200551	Neutral	Low	0.004	15.33	1	0.356955
*GNA11*	chr19:3115037 C>T	0.330139	Neutral	Low	0.043	13.43	1	0.106818
*SMO*	chr7:129209384 C>A	0.261754	Neutral	Low	0.429	12.79	1	0.0460526
*FGFR2*	chr10:121515246 G>A	0.641029	Oncogenic	Low	0.104	12.63	1	0.0643821
*NRAS*	chr1:114713886 T>C	0.505196	Oncogenic	Low	0.009	12.59	4	0.0503597–0.0715686
*MYC*	chr8:127738274 C>T	0.159967	Neutral	Low	0.131	12.48	1	0.626
*PIK3CA*	chr3:179204568 A>G	0.520249	Oncogenic	Low	0.027	12.35	1	0.162

**FIGURE 2:**
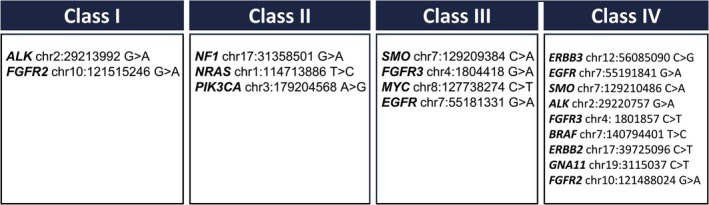
Synonymous variants of interest divided into four classes by support for possible pathogenicity. Class I variants are identified as potentially pathogenic by all three predictive algorithms (CScape, TraP, and CADD); Class II variants are identified by two algorithms, one of which is the cancer‐specific CScape algorithm; Class III variants are identified by both TraP and CADD algorithms, and Class IV variants are identified by a single algorithm.

We then classified variants identified as potentially pathogenic by CScape, TraP, and CADD into four categories based on the number and type of predictors that identified them as potentially pathogenic (Figure 2). This classification strategy was designed to integrate information from the algorithms and databases described above, allowing for a multi‐point decision‐making approach rather than relying on a single predictive tool.

No variants had a high enough prevalence within the cohort to be statistically assessed for impact on survival.

Variants in classes 1–3 were grouped based on the affected signaling pathways. The pathways most enriched with novel variants were MAPK/ERK and PI3K/AKT, with a total of four variants, each occurring once in the cohort (Table 5).

**TABLE 5:** Novel variants in MAPK/ERK and PI3K/AKT pathways with 2 or more predictions of pathogenicity from CScape, CADD, and TRaP.

Gene	Variant	Class	Cscape			TRaP score	CADD Phred‐like score	Patient TNM stage	Clinical variant present
			Score	Prediction	Confidence				
*ALK*	chr2:29213992 G>A	1	0.656541	Oncogenic	Low	0.427	18.47	4	PIK3CA
*FGFR2*	chr10:121515246 G>A	1	0.641029	Oncogenic	Low	0.068	16.03	1	ERBB2
*FGFR3*	chr4:1804418 G>A	3	0.148019	Neutral	Low	0.056	15.81	4	KRAS (G12C)
*EGFR*	chr7:55181331 G>A	3	0.158837	Neutral	Low	0.101	15.34	1	ROS1

## 4 Discussion

Our cohort is, in some ways, comparable to the general population of non‐squamous NSCLC patients in the Western world whose tumors are recommended to undergo sequencing as part of their care. The demographics of this cohort align with national and international statistics in terms of subtype, mean age at diagnosis, and treatment modality.

This cohort also has some notable distinguishing characteristics. This cohort is slightly more female dominant than expected when looking at the national incidence rate of NSCLC, likely reflecting the increased proportion of adenocarcinoma, both in women with NSCLC and in cases subjected to sequencing for clinical care [4, 44]. As our institution is a referral center for thoracic surgery, this cohort also has an over‐representation of early‐stage NSCLC. The amount of tissue available for sequencing is higher in patients who undergo tumor resection; as a result, there are patients with advanced stage disease who do not receive NGS because there is insufficient tissue, further enriching this cohort for patients with early‐stage disease. Finally, for part of the study period, our institution was undertaking routine sequencing of squamous NSCLC, which was not the standard of care at the time; this provided us with the opportunity to assess synonymous mutations in squamous tumors [45].

This study was conducted using a tumor‐only sequencing approach, which reflects current routine practice in many clinical diagnostic settings where matched normal tissue is not always available [45]. While tumor‐only sequencing enables efficient and clinically actionable genomic profiling, it does not allow for an unequivocal distinction between somatic and germline variants. As a result, some variants detected may represent germline alterations rather than true tumor‐acquired events. To mitigate this limitation, variants were evaluated using population and allele frequency as described in the methods.

Our cohort also has some variance from published data regarding the incidence of common genomic variants. The most common DNA mutations found within the cohort were *KRAS* (35.12%), *EGFR* (9.07%), *PIK3CA* (4.82%), and *BRAF* (4.53%). The prevalence of *KRAS* mutations in our cohort was numerically higher than that seen in other published cohorts [36, 37]. This highlights the value of examining regional variations in cancer genomics and could reflect the high smoking rate in our region [46], since KRAS mutation is strongly associated with smoking [47, 48]. The low prevalence of *PIK3CA* mutations and *BRAF* mutations in the cohort were consistent with the literature.

Some of our findings with respect to synonymous variants are noteworthy. One synonymous variant, an A‐to‐G transition in the *BRAF* gene (chr7:140801453), was observed eight times. This variant was not found in existing databases or published literature. This specific variant occurs in the region that encodes the zinc fingers of the *BRAF* protein within conserved region 1 (CR1) (UniProt: P15056). In the inactive state, CR1 inhibits the kinase domain of B‐Raf. Upon activation, Ras binds to the Ras‐binding domain of B‐Raf, releasing this inhibition. The zinc fingers then facilitate the docking of B‐Raf to the membrane, where they bind to lipids, helping localize B‐Raf to specific membrane regions. This localization is crucial for B‐Raf's activation and function in the signaling pathway [49, 50]. Synonymous mutations may affect mRNA stability or translational efficiency, leading to changes in protein expression levels [51, 52]. A recent study demonstrated that synonymous mutations can have a functional impact on human cell lines and impact protein expression [53]. In the case of BRAF, alterations in protein expression could potentially dysregulate the MAPK/ERK signaling pathway, contributing to uncontrolled cell proliferation; pre‐clinical functional studies would be needed to explore this hypothesis.

Analysis of synonymous variants with predicted splicing impact identified a single variant: a G‐to‐A transition within the *FGFR3* gene (chr4:1804418). Although absent from ClinVar, this variant received a significant acceptor loss score from SpliceAI indicating potential disruption of the splice acceptor site. *FGFR3* encodes the fibroblast growth factor receptor 3, a transmembrane receptor tyrosine‐protein kinase integral to regulating cell proliferation, differentiation, and apoptosis. FGFR3 is involved in both the MAPK/ERK and PI3K/AKT pathways, where it recruits proteins to activate RAS and PI3K, respectively. The variant is located between the exons encoding an immunoglobulin‐like domain—which is responsible for binding to fibroblast growth factors—and the exon encoding the protein kinase domain (UniProt: P22607). Loss of the splice acceptor site may lead to aberrant splicing patterns, resulting in the production of alternative mRNA isoforms. These isoforms may lack the sequences encoding the kinase domain or contain insertions/deletions that disrupt its proper structure or function. A variant that affects splicing in this region may lead to improper function of FGFR3, theoretically leading to abnormal cell proliferation and apoptosis, which are key characteristics of cancer.

One of the possible predicted pathogenic variants was also one of the top 10 most prevalent variants. This variant was a T to C transition in *NRAS* (chr1:114713886) that occurred four times in the cohort. The variant occurs just outside the effector region of the protein in a helix (UniProt: P01111). The location of the variant near the effector region suggests that it might affect mRNA secondary structure or regulatory elements important for translation or protein folding. Theoretically, structural alterations to the mRNA in this region can stabilize or destabilize mRNA based on the secondary structure, dependent on the secondary structure of the mRNA. If the mRNA is stabilized, this generally increases the cellular concentration of the mRNA as it slows the degradation rate [54, 55, 56]. Increased cellular concentration can result in increased protein expression, which may lead to hyperactivation of downstream signaling pathways, such as the MAPK/ERK pathway, promoting uncontrolled cell proliferation, survival, and metastasis.

Several novel variants were found in genes implicated in the MAPK/ERK and PI3K/AKT pathways, namely *ALK*, *FGFR2*, *FGFR3*, and *EGFR*. The PI3K/AKT pathway regulates cell survival and inhibits apoptosis [57] while the MAPK/ERK pathway regulates gene expression involved in proliferation, differentiation, and survival.

Like *FGFR3*, the *FGFR2* gene encodes a transmembrane receptor tyrosine‐protein kinase but is more widely expressed in various tissues. The synonymous *FGFR2* variant (chr10:121515246) identified is a G to A transition substitution. Like the *FGFR3* variant, this variant occurs between the domains that encode for the immunoglobulin‐like region that directly interacts with proteins in the extracellular region and the protein kinase domain (UniProt: P21802). This variant is not predicted to alter splicing. One of the ways this variant could have an impact is through codon usage bias. If this variant changes a codon to one that is less utilized in the cell, it could slow down translation or cause premature termination, potentially affecting protein folding and function. Alternatively, it could lead to a codon that is more utilized in the cell. Regardless, downregulation or upregulation of mRNA can both ultimately lead to increased expression either directly or through compensatory upregulation. Like *FGFR3*, a variant in *FGFR2* that may result in improper function of the protein would theoretically lead to abnormal cell proliferation and apoptosis, which again are key markers of cancer.

The other synonymous variant, supported by three pathogenicity prediction scores but no significant scores for splicing impact, is a G to A transition in the *ALK* gene (chr2:29213992). The *ALK* gene encodes a transmembrane receptor tyrosine kinase that, like the *FGFR* family, initiates signaling cascades in both the MAPK/ERK and PI3K/AKT pathways [58]. This variant occurs in the region of the gene that encodes for the protein kinase domain (UniProt: Q9UM73). Like the variants in the *FGFR* family, a variant in this region could alter mRNA secondary structure through codon usage bias—such as leading to protein truncation—potentially affecting the efficiency of translation of the ALK protein kinase domain.

The final of the four variants is in the *EGFR* gene which encodes for the epidermal growth factor receptor, a transmembrane receptor tyrosine kinase. Like many tyrosine kinases, EGFR dimerizes upon ligand binding and autophosphorylates, leading to recruitment of adaptor proteins and initiation of signaling cascades in MAPK/ERK and PI3K/AKT. This variant—a G to A transition—occurs in the region that encodes for the protein kinase domain (UniProt: P00533). Like the variant in *ALK*, which also encodes for the protein kinase domain, this variant has no predicted splicing impact. The possible impacts of this variant are similar to those mentioned for *ALK* above.

Although the limited size of the cohort does have its disadvantages, there also exist significant advantages. As a smaller province with only two referral centers for thoracic surgery and radiation oncology, this study is able to capture a significant portion of New Brunswickers, not only those who live in the city of Saint John. This includes rural patients who may have differing environmental exposures compared to their urban counterparts. The role of radon and other environmental exposures in lung cancer development is of particular interest in Canada. Compared to Sweden—a country with similar smoking rates and living conditions—Canada has a higher age‐adjusted annual incidence of lung cancer [59]. Environmental exposures like radon and arsenic are of particular interest, especially in Atlantic Canada, where over 40% of the population lives rurally, far above the national average of 17.8% [60]. Rural residents can experience as much as 31.2% greater average residential radon levels than urban counterparts, partially due to wells acting as conduits for radon exposure [61]. Arsenic is also found in hyperabundance in Atlantic Canada [5, 62].

Environmental carcinogens such as radon and asbestos are genotoxic—they directly damage DNA or cause genetic alterations, contributing to cancer development through mechanisms distinct from those associated with tobacco smoke [63, 64, 65]. We speculate that this could potentially lead to NSCLC mutation profiles in our region that are different from those seen in other regions, illustrating the value of examining the biology of lung cancers found in different parts of the world.

We present a list of synonymous variants with their associated predicted impact on splicing and possible pathogenicity. Although targeted therapy has been a significant breakthrough, there still exist areas for advancements. Due to the heterogeneous nature of the disease, many patients do not have targeted therapies available. We suggest that further work should focus on the nine variants in classes I, II, and III; particularly those involved in the MAPK/ERK and PI3K/AKT pathways. The MAPK/ERK and PI3K/AKT pathways are especially noteworthy because they play key roles in cell growth and survival, which are crucial functions in cancer progression. By exploring the role of synonymous mutations, there is the potential to enhance our understanding of cancer biology and potentially lead to novel strategies for treatment and patient management.

## 5 Conclusion

KRAS was the most common mutation in this cohort from Saint John, New Brunswick, with a higher prevalence than reported in other populations. Several novel synonymous variants were identified, including in *ALK*, *EGFR, FGFR2*, *FGFR3*, *MYC, NF1, NRAS*, and *PIK3CA*, with potential effects on MAPK/ERK and PI3K/AKT signaling. These findings suggest regional variation in NSCLC genomics and support further study of synonymous variants in disease progression.

## Author Contributions

**K.V.:** investigation, data curation, code development, formal analysis, methodology, writing – original draft, review, and editing. **L.M.:** code development, methodology, and writing – original draft, review, and editing. **D.I.:** supervision, investigation, and resources. **M.H., C.A., J.M., T.D.‐M., R.T., B.J., C.R.:** investigation and resources. **J.E.B.:** supervision, resources, and funding acquisition. **D.G.:** supervision, resources, and code development. **T.R.:** supervision, funding acquisition, conceptualization, resources, writing – review and editing.

## Conflicts of Interest

The authors declare no conflicts of interest.

## Data Availability

The data that support the findings of this study are available on request from the corresponding author. The data are not publicly available due to privacy or ethical restrictions.
